# Impact of intraamniotic inflammation on tryptophan metabolism in the placenta-fetal brain axis in rats

**DOI:** 10.1530/REP-24-0378

**Published:** 2025-04-18

**Authors:** Cilia Abad, Ivana Musilova, Eva Cifkova, Ramon Portillo, Fiona Kumnova, Rona Karahoda, Martin Sterba, Miroslav Lisa, Marian Kacerovsky, Jaroslav Stranik, Ales Stuchlik, Frantisek Staud

**Affiliations:** ^1^ Department of Pharmacology and Toxicology, Charles University, Faculty of Pharmacy in Hradec Kralove, Hradec Kralove, Czech Republic; ^2^ Department of Obstetrics and Gynecology, University Hospital Hradec Kralove, Charles University, Faculty of Medicine in Hradec Kralove, Hradec Kralove, Czech Republic; ^3^ Department of Chemistry, Faculty of Science, University of Hradec Kralove, Hradec Kralove, Czech Republic; ^4^ Department of Pharmacology, Charles University, Faculty of Medicine in Hradec Kralove, Hradec Kralove, Czech Republic; ^5^ Laboratory of Neurophysiology of Memory, Institute of physiology of the Czech Academy of Sciences, Prague, Czech Republic

**Keywords:** intrauterine inflammation, tryptophan metabolism, placenta, fetal brain, neurodevelopmental disorders

## Abstract

**In brief:**

Intrauterine inflammation disrupts tryptophan metabolism in both the placenta and the fetal brain, leading to a shift toward neurotoxic metabolites. These findings highlight the critical role of placental function in neurodevelopment and suggest that inflammation-induced metabolic changes may contribute to neurodevelopmental disorders.

**Abstract:**

The placenta plays a crucial role beyond nutrient transfer, acting as a dynamic endocrine organ that significantly influences maternal physiology and fetal development. It responds rapidly to even slight changes in the *in utero* environment to promote fetal survival. Disruptions in placental function are increasingly recognized as key contributors to the origins of neurodevelopmental disorders. In this study, we employed advanced technology to induce intrauterine inflammation through ultrasound-guided administration of LPS into gestational sacs. We then evaluated its effects on the gene expression of enzymes involved in TRP metabolism and conducted a comprehensive LC/MS analysis of the metabolome in the placenta and fetal brain of Wistar rats. Our results show that intraamniotic injection of LPS induces a robust inflammatory response leading to significant alterations in TRP metabolism, including downregulation of tryptophan hydroxylase (TPH) in the placenta, resulting in a decrease in serotonin (5-HT) levels. Similarly, in the fetal brain, exposure to LPS led to reduced *Tph* expression and increased monoamine oxidase expression, suggesting a decrease in 5-HT synthesis and an increase in its degradation. Furthermore, an upregulation of the kynurenine pathway was observed in both the placenta and fetal brain. Moreover, we detected a shift toward neurotoxicity, evidenced by an imbalance between neuroprotective and neurotoxic metabolites, including decreased levels of kynurenic acid and upregulation of kynurenine monooxygenase in the fetal brain. In conclusion, our findings reveal significant alterations in TRP metabolism following intrauterine inflammation, potentially contributing to neurodevelopmental disorders.

## Introduction

Intraamniotic inflammation is increasingly recognized as a significant contributor to the pathogenesis of spontaneous preterm birth (PTB). This inflammation can be due to microbial invasion of the amniotic cavity (MIAC), leading to intraamniotic infection or due to activation of sterile intraamniotic inflammation by damage-associated molecular patterns (Romero *et al.* 2014*a*,*b*). Notably, infection and inflammation are documented as primary risk factors for PTB, with intraamniotic infection identified in at least 25% of cases (Cappelletti *et al.* 2016). This is accompanied by an increased production of proinflammatory cytokines, which are strongly associated with uterine activation and the onset of PTB. IL-1β, TNF and IL-6 are critical cytokines implicated in this process, with IL-6 recently identified as a key marker of intraamniotic inflammation and a predictor of PTB (Goepfert *et al.* 2001).

The impact of prenatal inflammation extends beyond pregnancy, as it is closely related to adverse neurodevelopmental, psychiatric, cognitive and behavioral outcomes in the offspring. Maternal bacterial and viral infections during pregnancy have been shown to significantly increase the risk of neuropsychiatric disorders such as schizophrenia, autism spectrum disorders and cognitive impairments (Elovitz *et al.* 2011, Kwon *et al.* 2022). Despite the established association, the precise mechanisms by which inflammation influences fetal brain development and programming remain largely unclear. Emerging evidence, however, suggests that the placenta plays a central role in mediating these effects, particularly through its response to intrauterine stressors such as inflammation. This has led to the development of the ‘placenta–brain axis’ concept, which highlights the critical interaction between placental function and fetal brain development within the broader framework of the Developmental Origins of Health and Disease (DOHaD) concept (Rosenfeld 2021).

**Figure 1:**
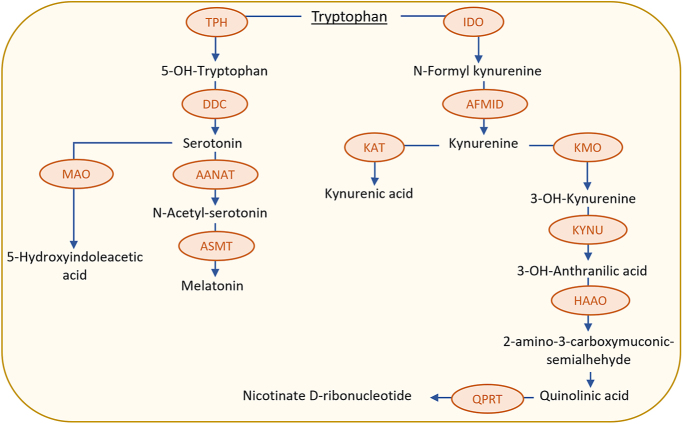
Tryptophan metabolic pathway. TRP is metabolized by two main pathways: the serotonin pathway and the kynurenine pathway. The main catabolic pathways of TRP are shown with the enzyme metabolizing the corresponding catabolites. TPH, tryptophan hydroxylase; DDC, tryptophan decarboxylase; AANAT, aralkylamine N-acetyltransferase; ASMT, N-acetylserotonin O-methyltransferase; MAO, monoamine oxidase; IDO, indoleamine 2,3-dioxygenase; AFMID, arylformamidase; KAT, kynurenine amino transferase; KMO, kynurenine 3-monooxygenase; KYNU, kynureninase; HAAO, 3-hydroxyanthranilate 3,4-dioxygenase; QPRT, quinolinate phosphoribosyltransferase.

In recent years, placental L-tryptophan (TRP) metabolism has emerged as a potential mechanistic link between prenatal inflammation and the increased risk of mental and cognitive disorders in later life. This connection is attributed to the neuroactive properties of several TRP metabolites (Bonnin & Levitt 2011, Goeden *et al.* 2013). In both the placenta and the fetal brain, two main TRP metabolic pathways have been identified: the kynurenine (KYN) and serotonin (5-HT) pathways (Xue *et al.* 2023) (Fig. 1). TRP catabolism via the KYN pathway produces metabolites such as KYN, kynurenic acid (KYNA), 3-hydroxykynurenine and quinolinic acid (QUIN) with neuroactive, antioxidant and immunoregulatory properties (Badawy 2017). TRP metabolism via the 5-HT pathway generates essential metabolites, including 5-HT (crucial for fetal brain development (Bonnin *et al.* 2011)) and melatonin (Peric *et al.* 2022).

Both TRP metabolic pathways are highly responsive to inflammatory stimuli. In particular, intrauterine infections have been associated with the upregulation of genes encoding KYN pathway enzymes in the placenta (Manuelpillai *et al.* 2005). In a well-characterized clinical cohort, we recently identified significant impairments in TRP metabolism-related gene expression in the placenta associated with PTB; these changes showed a positive correlation with inflammatory markers in both intraamniotic fluid and maternal circulation (Karahoda *et al.* 2021). In addition, within the same cohort, we observed substantial metabolic alterations in the placenta, particularly in amino acid availability (including reduced leucine, glutamine and tyrosine levels), purine metabolism and tryptophan catabolism, all of which are critical for fetal developmental programming (Cifkova *et al.* 2024). Furthermore, using *ex vivo* models of human placenta explants, we have recently demonstrated that exposure to LPS or poly I:C disrupts TRP homeostasis in the human term placenta. This disruption is characterized by decreased 5-HT production during intrauterine life and an imbalance in KYN metabolites, potentially leading to adverse effects on fetal brain development and programming (Abad *et al.* 2024).

Ethical and technical constraints limit experiments in pregnant women, making animal models indispensable for investigating intraamniotic inflammatory complications in PTB due to their ability to support diverse study designs and provide comprehensive insights into the pathogenesis of intraamniotic inflammation (Nielsen *et al.* 2016). Several species, including sheep, non-human primates and rodents, have been used to model intraamniotic inflammation following LPS exposure (Kallapur *et al.* 2009, Kramer *et al.* 2009, Jackson *et al.* 2022, Presicce *et al.* 2024). While large animal models such as sheep and primates provide physiological similarities to human pregnancy, rodent models offer key advantages in terms of cost-effectiveness, experimental flexibility and genetic manipulation (Stranik *et al.* 2022). Recently, we have established a sophisticated rat model of intraamniotic inflammation (Stranik *et al.* 2022), using ultrasound-guided transabdominal administration of LPS into individual gestational sacs. This approach has two main benefits: first, administering an intraamniotic trigger agent effectively induces a localized inflammatory response characterized by increased IL-6 levels in the amniotic fluid, facilitating detailed studies of intraamniotic complications and simulating various clinical scenarios. Second, Wistar rat provides an ideal model for investigating placental tryptophan (TRP) metabolism in both health and disease contexts (Badawy 2015).

In this study, utilizing our established rat model of ultrasound-guided intraamniotic injection, we aimed to investigate the effects of LPS-induced inflammation on the gene expression of enzymes involved in TRP metabolism. In addition, we conducted a comprehensive metabolomic analysis of the placenta and fetal brain to better understand the biochemical interactions triggered by intraamniotic inflammation and their impact on pregnancy and fetal development.

## Materials and methods

### Animals

The experiments were performed using pregnant female Wistar rats purchased from Velaz (Czech Republic). All experimental procedures were conducted in compliance with the Act on the Protection of Animals against Cruelty (Act No. 246/1992 Coll.), with approval from the Animal Welfare Committee of the Faculty of Medicine in Hradec Kralove, Charles University and the Czech Ministry of Education, Youth and Sports (No. 41058/2016-MZE-1721). The pregnant rats were maintained in cages under constant room temperature (22 ± 2°C), with relative air humidity of 50 ± 10%, low noise levels and standard 12 h light:12 h darkness cycles (12L:12D) in the vivarium of the Faculty of Medicine at Hradec Kralove. All rats received food and water *ad libitum*. Gestation day 1 (GD1) was designated as the morning when vaginal plug formation was observed. A total of eight pregnant rats were used in this study, with four assigned to the control group and four to the treatment group.

### Ultrasound-guided intraamniotic injections

The intraamniotic injections were performed according to the protocol previously published, using the same cohort of animals described in the original article (Stranik *et al.* 2022). In brief, on GD18, dams were anesthetized with isoflurane, initially in an induction chamber with 5% isoflurane in oxygen, followed by maintenance anesthesia with 1.5–2.0% isoflurane and a 2 L/min oxygen flow. The animals were positioned securely on a heated pad provided by the Vevo Imaging Station (FUJIFILM VisualSonics Inc., Canada). Body temperature was maintained within the range of 37 ± 1°C and monitored using a rectal thermometer (FUJIFILM VisualSonics Inc., Canada). Heart rate and respiratory rate were monitored using electrodes integrated into the heating pad. Fur removal was accomplished using a depilatory cream. The ultrasound transducer MX400, connected to the ultrasonographic system Vevo 3100 (both from FUJIFILM VisualSonics Inc., Canada), was positioned and stabilized using a mechanical holder on the Vevo Imaging Station to focus on the targeted gestational sac. Guided by ultrasound imaging, 10 μg of LPS (E. coli, serotype O55:B5, Sigma-Aldrich, Czechia) dissolved in 100 μL of phosphate-buffered sterile saline (PBS) was administered intraamniotically using a 27 G × 40 mm needle and an Omnican® 50 syringe, secured in a mechanical holder. Control animals received injections of 100 μL of sterile PBS. In each animal from both groups, approximately 34% of amniotic sacs were injected. Manipulation was limited to accessible gestational sacs with accurate positional recordings (Stranik *et al.* 2022). After the procedure, the animals were allowed to recover under a heat lamp before being returned to their respective cages.

### Placenta and fetal brain sample collection

Twenty-four hours following the intraamniotic administration of LPS, on GD19, animals were anesthetized with isoflurane and prepared for ultrasound examination using the same protocol as described above for the initial administration. The position of the gestational sac and the vitality of the offspring were evaluated using the MX250S ultrasound transducer (15–30 MHz). Surgical anesthesia was then achieved (isoflurane 3.5%), and a midline abdominal incision was made to expose the uterine horns. Subsequently, both uterine horns were excised from the abdominal cavity, and placentas as well as fetal brains were collected from four injected sacs per animal. The collected tissues were snap-frozen and stored at −80°C for subsequent analyses. The animals were euthanized by anesthesia overdose.

### RNA isolation and reverse transcription (RT)

Total RNA extraction from weighed tissue samples was conducted using Tri Reagent solution following the manufacturer’s guidelines. RNA purity was assessed using the A260/A280 ratio, while contamination by organic solvents was evaluated using the A260/230 ratio. Measurements were taken with a NanoDrop™ 1000 Spectrophotometer (Thermo Fisher Scientific, USA), with the A260 value used to determine total RNA concentration. Subsequently, RT was carried out using the iScript Advanced cDNA Synthesis Kit and T100 Thermal Cycler (Bio-Rad, USA).

### Quantitative PCR analysis

Gene expression analysis in rat placenta and fetal brain was conducted using the QuantStudio™ 6 (Thermo Fisher Scientific, USA) for quantitative PCR (qPCR). Each reaction contained cDNA (12.5 ng/μL) and was performed in a total reaction volume of 5 μL per well. TaqMan® Universal Master Mix II without UNG (Thermo Fisher Scientific, USA) and pre-designed TaqMan® Real-Time Expression PCR assays were utilized. Triplicate amplifications were carried out for each sample following the thermal program specified in the manufacturer’s instructions. Relative gene expression was normalized to the geometric mean of β2-microglobulin (*B2m*), tyrosine 3-monooxygenase/tryptophan 5-monooxygenase activation protein zeta (*Ywhaz*) and glyceraldehyde-3-phosphate dehydrogenase (*Gapdh*) as reference genes for placenta analysis and to *B2m*, *Ywhaz*, *Gapdh* and TATA-box binding protein (*Tbp*) as reference genes for fetal brain analysis.

### LC/MS metabolomic analysis

Approximately 10 mg of placenta or fetal brain tissue was homogenized in an ice-cold methanol – 0.1% aqueous formic acid (4:1, v/v) mixture (180 μL/mg of tissue) with zirconium oxide beads (SiLibeads, Germany) and Garnet matrix (MP Biomedicals, USA) (both 24 mg/mg of tissue) using a FastPrep-24™ 5G (MP Biomedicals) in 3 × 30 s cycles with 30 s pauses on an ice bath at a device speed of 6 m/s. The mixture was centrifuged for 30 min at 16,873 ***g*** and 1.5 mL of supernatant was collected, evaporated to dryness using a vacuum concentrator (Eppendorf, Germany) and redissolved in a 40 μL acetonitrile – propan-2-ol – 0.2% aqueous formic acid (1:1:8, v/v/v) mixture before LC/MS analysis. The quality control (QC) sample was prepared by pooling equal aliquots of supernatants from the analyzed samples using the same extraction procedure. The QC sample was regularly injected within the sample set (after every ten injections).

The LC/MS metabolomic analysis was performed using an Acquity I-Class UPLC system and Vion IMS QqTOF mass spectrometer (Waters, USA) equipped with an Acquity UPLC HSS T3 column (150 × 2.1 mm, 1.8 μm, Waters) at a flow rate of 0.3 mL/min, an autosampler temperature of 8°C, a column temperature of 30°C and a gradient of the mobile phase: 0 min – 100% A, 2 min – 94% A, 3 min – 90% A, 7 min – 0% A, 7.7 min – 0% A, 10.5 min – 0% A at 0.55 mL/min, 12 min – 100% A, 18 min – 100% A, where A was 0.2% aqueous formic acid and B was acetonitrile – propan-2-ol (1:1, v/v). Electrospray ionization full-scan mass spectra were acquired in positive-ion mode with the following tuning parameters: mass range 50–1,000, soft transition mode, scan time 0.2 s, capillary voltage 0.5 kV, cone voltage 10 V, source offset 50 V, source temperature 100°C, desolvation temperature 600°C, cone gas flow 50 L/h and desolvation gas flow 800 L/h. Leucine enkephalin was used as the lock mass for all experiments.

Analyzed metabolites (Supplementary Table I (see section on Supplementary materials given at the end of the article)) were annotated in the pooled sample based on accurate m/z in full-scan mass spectra and fragment ions in tandem mass spectra acquired in ion mobility mode or identified using identical standards. The data were processed using Unifi software (Waters) with a mass tolerance of 7 ppm and the peak areas of individual metabolites were normalized to the corresponding metabolites in the closest QC sample, i.e. *A*_sample_/*A*_QC_.

### Statistical analysis

Experimental outcomes were analyzed using a Linear Mixed Model (LMM) to account for litter as a random effect, ensuring appropriate nesting of samples within each dam. The statistical analyses were performed using Python and estimated marginal means (EMMs) were extracted from the LMM model for group comparisons. Pearson correlations were used to assess relationships between variables. For visualization, fold changes were calculated as the ratio of the differences between the group means (LPS vs PBS). Statistical significance levels in the figures are indicated as follows: *P ≤ 0.05, **P ≤ 0.01 and ***P ≤ 0.001.

## Results

### Gene expression of proinflammatory cytokines in rat placenta and fetal brain exposed to intraamniotic LPS

**Figure 2:**
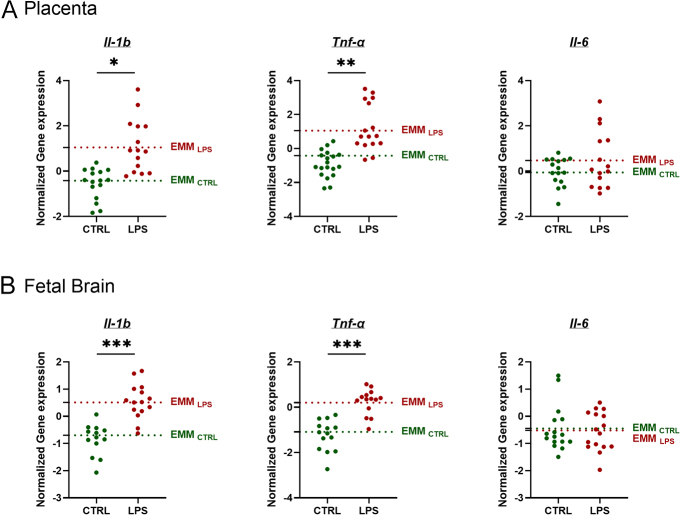
Gene expression of proinflammatory cytokines in rat placenta and fetal brain exposed to intraamniotic LPS. Proinflammatory cytokines *Il-1b*, *Tnf-α* and *Il-6* from rat placenta (A) and fetal brain (B) exposed to 10 μg LPS were analyzed by qPCR. Data are presented as individual points, with EMMs shown as dotted lines; *n* = 4 dams per group. Statistical significance was evaluated using a LMM to account for litter effects; *P ≤ 0.05, **P ≤ 0.01 and ***P ≤ 0.0001.

Exposure of the intraamniotic environment to LPS (10 μg) significantly increased the gene expression of the proinflammatory cytokines *Il-1b* and *Tnf-α* after 24 h in both the placenta and the fetal brain, as shown in Fig. 2A and B. No significant changes in *Il-6* gene expression were observed in the placenta or fetal brain after 24 h of exposure to LPS compared to the control.

### Intraamniotic inflammation regulates the gene expression of enzymes involved in TRP metabolic pathways in the rat placenta and fetal brain

**Figure 3:**
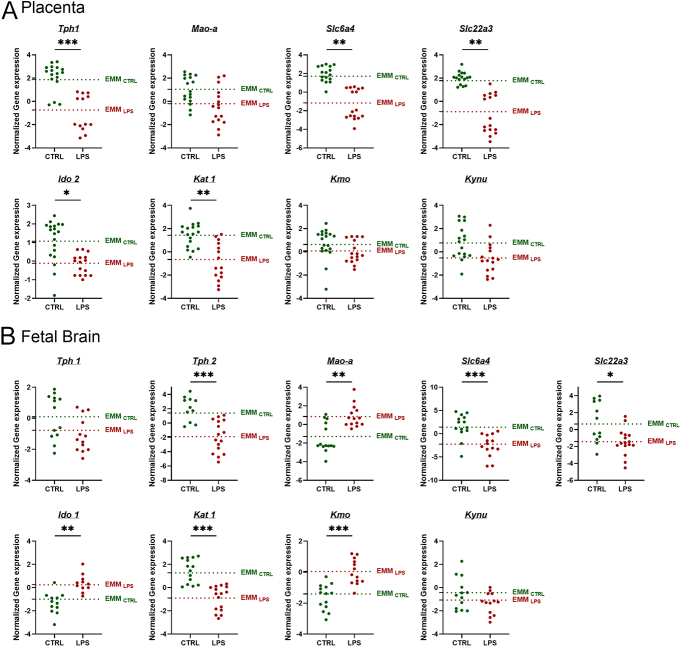
Gene expression of the main enzymes and transporters involved in TRP metabolic pathways in rat placenta and fetal brain exposed to intraamniotic LPS. Enzymes and transporters of the 5-HT pathway (*Tph1, Tph2, Mao-a, Scl6a4* and *Scl22a3)* and enzymes of the KYN pathway (*Ido*, *Kat1*, *Kmo* and *Kynu)* from rat placenta (A) and fetal brain (B) exposed to intraamniotic LPS (10 μg) were analyzed by qPCR. Data are presented as individual points, with EMMs shown as dotted lines; *n* = 4 dams per group. Statistical significance was evaluated using a LMM to account for litter effects; *P ≤ 0.05, **P ≤ 0.01 and ***P ≤ 0.001.

The effects of 24 h exposure to intraamniotic LPS (10 μg) on the expression of key genes associated with TRP metabolism in the rat placenta and fetal brain were evaluated using qPCR. As shown in Fig. 3A, after LPS-induced intraamniotic inflammation, the placenta exhibited downregulation of *Tph1*, suggesting a decrease in TRP metabolism through the 5-HT pathway. Furthermore, *Slc6a4* (*Sert*) and *Slc22a3* (*Oct3*), the transporters responsible for placental uptake and clearance of serotonin, were also downregulated, indicating a potential disruption in serotonin homeostasis and availability. In addition, downregulation of *Ido2* and *Kat1* was observed while *Kmo* and *Kynu* were not affected by intraamniotic inflammation.

In the fetal brain, on the other hand (Fig. 3B), downregulation of *Tph2* and upregulation of *Mao-a* were observed following LPS-induced intraamniotic inflammation, whereas T*ph1* expression remained unaffected. In addition, both *Slc6a4* (*Sert*) and *Slc22a3* (*Oct3*) were downregulated in response to LPS exposure. The first and rate-limiting enzyme of the KYN pathway, *Ido1*, was upregulated, suggesting preferential TRP metabolism via the KYN pathway under inflammatory conditions. Furthermore, downregulation of *Kat1* and upregulation of *Kmo* were observed, possibly favoring the metabolism of KYN to neurotoxic QUIN rather than neuroprotective KYNA.

**Figure 4:**
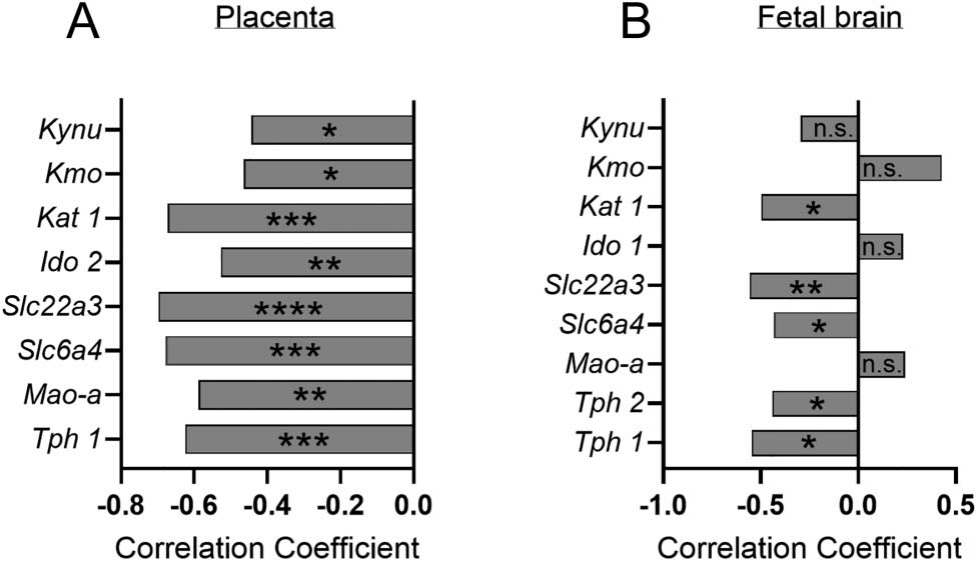
Relationship between amniotic fluid IL-6 concentration and gene expression of the main enzyme involved in the 5-HT and KYN pathways of TRP metabolism. Pearson correlation between relative gene expression and amniotic fluid IL-6 concentration in the placenta (A) and fetal brain (B) after 24 h of exposure to intraamniotic LPS (10 µg). Normalized gene expression data were log2-transformed *P ≤ 0.05 and **P ≤ 0.01.

Although *Il-6* gene expression was not altered after 24 h of exposure to LPS in this study, our previous research, in which we established the model and measured amniotic fluid concentrations from the same cohort, demonstrated a significant increase in IL-6 concentrations in amniotic fluid following intraamniotic exposure to LPS (Stranik *et al.* 2022). This prior data on amniotic fluid IL-6 concentrations, presented in our original paper, has been utilized in the current study to correlate with the gene expression to better understand the effects of intraamniotic inflammation on TRP genes. The Pearson correlation test was applied to identify genes whose expression was correlated with IL-6 concentrations in the amniotic fluid. Of the genes tested in the placenta, the relative expressions of *Tph1* (*r* = −0.6249, *P* = 0.0006), *Mao-a* (*r* = −0.5877, *P* = 0.0010), *Slc6a4* (*r* = −0.6781, *P* = 0.0001), *Slc22a3* (*r* = −0.6980, *P* = 0.0001), *Ido2* (*r* = −0.5270, *P* = 0.0033), *Kmo* (*r* = −0.4646, *P* = 0.0111), *Kynu* (*r* = −0.4445, *P* = 0.0202) and *Kat1* (*r* = −0.6731, *P* = 0.0001) were significantly negatively correlated with amniotic fluid IL-6 concentrations (Fig. 4A). Furthermore, among the genes evaluated in the fetal brain, *Tph1* (*r* = −0.5467, *P* = 0.0126), *Tph2* (*r* = −0.4407, *P* = 0.0401), *Slc6a4* (*r* = −0.4334, *P* = 0.0439), *Slc22a3* (*r* = −0.5586, *P* = 0.0037) and *Kat1* (*r* = −0.4978, *P* = 0.0115) showed a significant negative correlation with IL-6 concentrations (Fig. 4B). The original correlation graphs between IL-6 concentration in amniotic fluid and normalized gene expression in the placenta and fetal brain are presented in Supplementary Figs 1 and 2, respectively.

### Intraamniotic inflammation modulates the placenta and fetal brain metabolites

**Figure 5:**
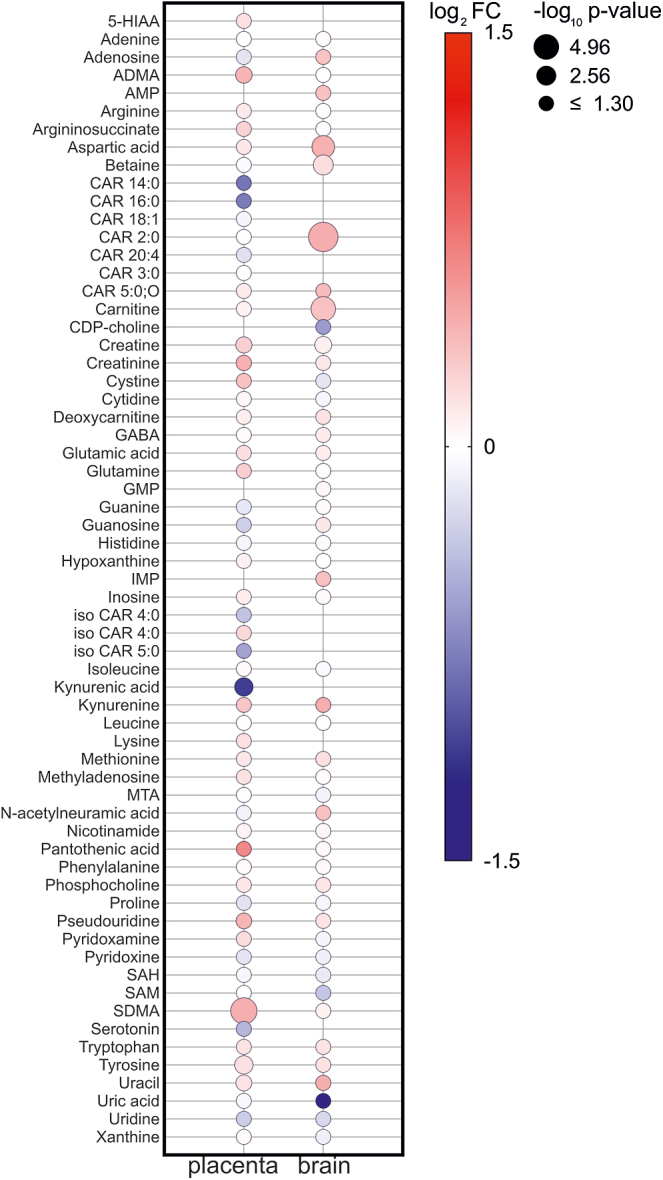
Changes in the placental and fetal brain metabolomic profile in LPS-triggered intraamniotic inflammation. Bubble plot highlighting metabolites that are dysregulated in placentas and brain exposed to intraamniotic LPS compared to PBS. The node color gradient indicates the difference in metabolite levels between LPS and PBS, expressed as a fold change, while the node radius reflects the negative log of the *P*-value.

A total of 59 metabolites were identified in the placenta and 53 metabolites in the fetal brain using LC/MS. Figure 5 illustrates the fold changes (LPS vs PBS intraamniotic injection) in the placenta and fetal brain for all identified metabolites. In placental tissue, 11 of the 59 metabolites showed significant changes after 24 h of intrauterine inflammation. The most pronounced changes were observed in metabolites related to TRP metabolism (27%) and other key metabolic pathways. In the placenta, significant alterations were detected in kynurenine, kynurenic acid and serotonin, alongside metabolites involved in creatine metabolism (creatine and creatinine), pyrimidine metabolism (pseudouridine and uracil), nitric oxide synthesis (ADMA and SDMA) and vitamin metabolism (pantothenic acid), as well as the amino acid tyrosine. In the fetal brain, six of the 53 metabolites displayed significant differences between intraamniotic injections of LPS and PBS. Although metabolic changes were less pronounced compared to the placenta, significant alterations were observed in aspartic acid, betaine, carnitine, Car2:0, creatine and uracil following intraamniotic LPS exposure. These findings suggest a broader metabolic shift affecting neurotransmitter pathways, energy metabolism and nucleotide turnover in response to inflammation.

**Figure 6:**
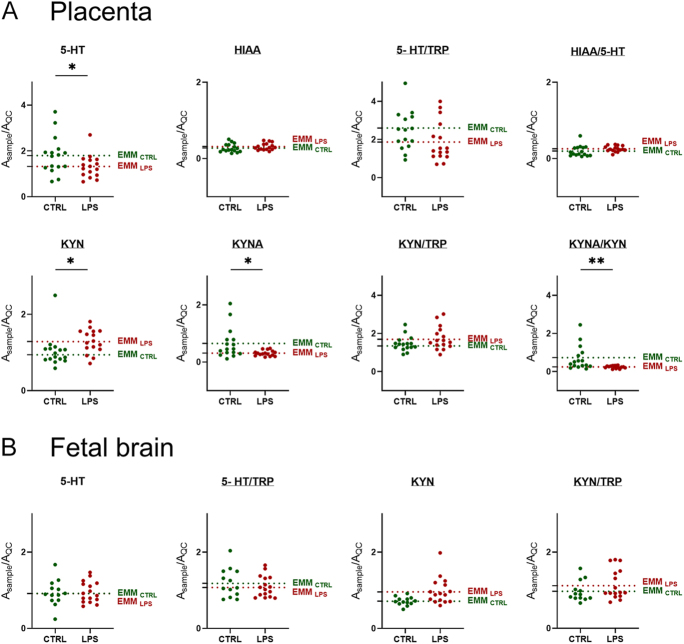
Metabolites and product/substrate ratios related to TRP metabolism in rat placenta and fetal brain. Graphs showing differences between LPS- and PBS-treated rats for metabolites related to the 5-HT and KYN pathways in the placenta (A) and fetal brain (B). Data are presented as individual points, with EMMs shown as dotted lines; *n* = 4 dams per group. Statistical analysis was performed using a LMM to account for litter. *P ≤ 0.05 and **P ≤ 0.01.

In line with our hypothesis that changes in tryptophan metabolism could explain the relationship between intrauterine inflammation and neurodevelopmental disorders, we focused on metabolites involved in both the serotonin and kynurenine pathways of TRP metabolism, as shown in Fig. 6. In the placenta, LPS-induced intrauterine inflammation led to a decrease in 5-HT and an increase in KYN concentrations, suggesting a shift toward TRP catabolism through the kynurenine pathway in an intraamniotic inflammatory environment. However, HIAA levels, as well as the HIAA/5-HT, 5-HT/TRP and KYN/TRP ratios, remained unchanged. In addition, KYNA levels decreased in the placenta following intrauterine inflammation, accompanied by a reduction in the KYNA/KYN ratio, suggesting reduced KYN catabolism through the KAT enzyme. In the fetal brain, 5-HT and KYN concentrations remained unchanged, while HIAA and KYNA were not detected. No significant variations were observed in TRP concentrations in the placenta or fetal brain after intraamniotic injections of LPS (Supplementary Fig. 3).

## Discussion

In most mammalian species, the placenta – a temporary yet complex and diverse organ – serves as the critical interface for the exchange of gases, nutrients and waste products between mother and fetus. Beyond its role in resource transfer, the placenta synthesizes hormones and metabolites that profoundly influence both maternal physiology and fetal development (Bowman *et al.* 2021). The placenta swiftly responds to even slight changes in the *in utero* environment to promote fetal survival. These responses can alter fetal developmental trajectories, particularly in the brain, which is highly vulnerable to placental disruptions. Neurobehavioral disorders often trace their origins to pathophysiological changes in the placenta (Marsit *et al.* 2012, Paquette *et al.* 2013), underscoring the critical interdependence between placental function and fetal brain development (Rosenfeld 2021). In this study, we employed advanced technology to induce intrauterine inflammation through ultrasound-guided administration of LPS into gestational sacs. We then evaluated its effects on the gene expression of enzymes involved in TRP metabolism and conducted a comprehensive LC/MS analysis of the metabolome in the placenta and fetal brain of Wistar rats.

LPS, a Toll-like receptor 4 (TLR4) agonist that mimics bacterial infection, triggers signal transduction and activates transcription factors such as nuclear factor kappa B (NF-κB). This activation leads to the transcription of proinflammatory mediators, including cytokines, chemokines and complement proteins (Guijarro-Muñoz *et al.* 2014). Twenty-four hours after the intrauterine injection of LPS, we observed upregulation of the genes coding for the proinflammatory cytokines *Il-1b* and *Tnf-α* in both the placenta and fetal brain. Our findings are consistent with other studies showing that intrauterine stimuli result in increased concentrations of TNF-α and IL-6 in the placenta and fetal brain (Bell *et al.* 2004). LPS administration is a well-established inflammatory stimulus across various experimental models. Our results align closely with studies using intraperitoneal LPS injection in rats, where maternal exposure to LPS induces a time-dependent increase in *Il-1b* and *Tnf-α* mRNA expression in the fetal brain, detectable between 1 and 24 h (Cai *et al.* 2000). Another study using maternal intraperitoneal poly I:C injection in rats demonstrated upregulation of *Tnf-α* and *Il-6* in the placenta and fetal brain 6 h following systemic injection but not at the 24 h mark (McColl & Piquette-Miller 2019). This highlights the critical importance of timing when collecting samples to observe the genetic upregulation of proinflammatory cytokines and may explain the absence of changes in *Il-6* gene expression in the placenta and fetal brain after 24 h in our study. All these studies, including ours, confirm that LPS injection in pregnant animals initiates a significant inflammatory response in the fetus, leading to the production of proinflammatory cytokines within the fetal brain. Cytokines play a critical role as signaling molecules during normal brain development. Disruptions in cytokine balance or neuroinflammation can therefore have profound effects on neurodevelopment, potentially heightening susceptibility to neuropsychiatric traits (Woods *et al.* 2023).

Cytokines and TRP metabolism are closely associated. TRP metabolism contributes to the regulation of the immune response, the generation of oxidative radicals and the production of neuroregulatory substances (Moroni 1999, Xu *et al.* 2017). In addition, TRP metabolism is highly susceptible to proinflammatory cytokines (Kindler *et al.* 2020). We have previously demonstrated in human placenta explants that an inflammatory environment directly affects the 5-HT pathway by decreasing TPH (gene, protein and function) and reducing placental 5-HT levels (Abad *et al.* 2024). Similarly, inducing intrauterine inflammation resulted in downregulation of *Tph1* in the placenta, accompanied by a decrease in 5-HT levels. Consistent with our findings, intrauterine inflammation led to reduced 5-HT levels in the placenta and brains of newborn rabbits (Kannan *et al.* 2011, Williams *et al.* 2017). In the placenta, 5-HT can exert autocrine and paracrine effects by binding to and activating membrane-bound 5-HT receptors (Viau *et al.* 2009). Placental 5-HT insufficiency has been associated with autism spectrum disorders (Sato 2013, Yang *et al.* 2014) and anxiogenic behaviors (Beaulieu *et al.* 2008). Animal model studies further support that a precise balance of 5-HT signaling within the placenta is essential to maintaining its normal structure and functions (Laurent *et al.* 2016). The fetal brain is particularly vulnerable to fluctuations in placental 5-HT, especially since, during early brain development, the placenta is the sole source of this neurotransmitter (Bonnin *et al.* 2011, Bonnin & Levitt 2011). By GD19, the fetal brain is fully capable of synthesizing 5-HT through TRP metabolism (Abad *et al.* 2020). Beyond TPH and MAO, the placenta employs other mechanisms to regulate 5-HT homeostasis. It expresses membrane transporters for 5-HT uptake on both sides of the polarized trophoblast cells: the high-affinity, low-capacity serotonin transporter (SERT/SLC6A4) on the maternal side (Prasad *et al.* 1996) and the low-affinity, high-capacity organic cation transporter (OCT3/SLC22A3) (Karahoda *et al.* 2020*b*) on the fetal side. Previously, we highlighted the crucial role of these transporters in maintaining placental 5-HT balance at term (Karahoda *et al.* 2020*b*, Staud *et al.* 2023). In the present study, we observed downregulation of *Sert* and *Oct3* in the placenta following intraamniotic LPS exposure, suggesting a disruption in serotonin uptake and availability at the maternal–fetal interface under inflammatory conditions. Intrauterine inflammation also affects the 5-HT pathway in the fetal brain, as evidenced by the downregulation of *Tph2* and upregulation of *Mao*, suggesting a decrease in 5-HT synthesis coupled with an increase in its degradation. However, despite these gene expression changes, 5-HT levels remained unchanged, indicating possible compensatory mechanisms or time-dependent metabolic shifts that may not yet be reflected at the 24 h sampling point. The observed downregulation of *Sert* and *Oct3* in the fetal brain suggests a reduction in serotonin reuptake, which may contribute to maintaining extracellular serotonin levels by limiting its clearance from the synaptic space. In addition, whole-brain homogenization may mask region-specific differences, where certain brain areas may experience 5-HT depletion while others compensate to preserve overall homeostasis. During fetal brain development, 5-HT promotes cell division, neural migration, cell differentiation and synaptogenesis. Hyposerotonemia can affect sensory, motor and cognitive abilities, potentially leading to autism spectrum disorder or other neurobehavioral disorders (Yang *et al.* 2014).

In various organs including the placenta, proinflammatory cytokines have been widely documented to transcriptionally induce *IDO1* (Williams *et al.* 2017), increasing its protein levels and function (Abad *et al.* 2024). However, we have reported that *Ido1* is not expressed in the rat placenta; instead, *Ido2* is the predominant isoform expressed (Abad *et al.* 2020). The physiological function of *Ido*2 remains unclear and, unlike *Ido1*, its expression is not upregulated by the proinflammatory cytokine IFN-γ (Kudo *et al.* 2020). Interestingly, we observed that the intrauterine inflammatory environment led to decreased *Ido2* expression in the placenta. Further studies are required to identify the specific role of *Ido2* in the placenta. Despite the lack of *Ido1* expression and the absence of *Ido2* upregulation by proinflammatory cytokines, we observed an increase in KYN levels. This suggests that alternative proteins such as TDO may compensate for the absence or low activity of IDO (Karahoda *et al.* 2020*a*), explaining the elevated KYN levels. This finding aligns with other studies that demonstrate elevated KYN levels and activation of the KYN pathway following inflammatory stimuli (Kruse *et al.* 2019, Kindler *et al.* 2020).

Following LPS-induced intraamniotic inflammation, *Ido1* was upregulated in the fetal brain, suggesting enhanced TRP catabolism via the KYN pathway. Similar results have been observed in rabbits subjected to LPS-induced intrauterine inflammation, where *Ido1* was also upregulated, accompanied by increased KYN levels (Williams *et al.* 2017). However, in our study, despite the upregulation of *Ido1*, KYN levels remained unchanged. This difference may reflect species-specific variations, differences in sampling time or compensatory mechanisms that regulate KYN metabolism in the rat fetal brain. Activation of the KYN pathway during neuroinflammation can produce several neuroactive metabolites that may affect brain functions and behaviors. Animal studies have shown that continuous KYN supplementation to dams during the prenatal and postnatal periods causes memory impairments in adult offspring (Pocivavsek *et al.* 2012). Furthermore, intracerebroventricular administration of LPS in mice triggered depressive symptoms associated with induction of the KYN pathway in the brain (Lawson *et al.* 2013). Recent studies have identified cerebrospinal fluid levels of neopterin, KYN, QUIN and the KYN/TRP ratio as valuable biomarkers of neuroinflammation (Yan *et al.* 2023), providing additional insights into the role of TRP metabolism in neurological disorders. Several neuroactive metabolites are produced downstream in the KYN pathway, including the NMDA receptor agonist QUIN and, in a competing branch, KYNA, which antagonizes α7 nicotinic acetylcholine and the NMDA receptor. LPS-induced intraamniotic inflammation can not only induce KYN formation but also disrupt the balance between QUIN and KYNA. In this context, an intrauterine inflammatory environment downregulates K*at 1* in both the placenta and fetal brain. This leads to a reduction in KYNA levels and the KYNA/KYN ratio in the placenta, suggesting decreased KYN metabolism through the KAT branch. Furthermore, the upregulation of K*mo* in the fetal brain indicates enhanced KYN metabolism toward QUIN synthesis. Notably, the upregulation of the branch leading to neurotoxic metabolites such as QUIN and the downregulation of neuroprotective metabolites such as KYNA highlight a significant shift toward neurotoxicity (Pathak *et al.* 2024). These metabolic changes suggest a potential mechanism by which prenatal inflammation may impair fetal brain development and programming (Woods *et al.* 2023).

Our study, employing advanced LC/MS techniques, delineates metabolomic changes in response to intraamniotic inflammation, offering a detailed landscape of metabolic perturbations. While our primary focus was on metabolites related to TRP metabolism, we also observed significant changes in other metabolic pathways influenced by intraamniotic inflammation. In the placenta, inflammation led to increased levels of ADMA and SDMA, methylated arginine derivatives involved in nitric oxide regulation, suggesting endothelial dysfunction and altered vascular tone (Böger 2006), which may impact placental blood flow and fetal nutrient exchange. Similarly, elevated creatine and creatinine indicate disruptions in energy metabolism (Wyss & Kaddurah-Daouk 2000), while increased pseudouridine and uracil suggest enhanced RNA turnover in response to cellular stress (Rodell *et al.* 2024). Higher pantothenic acid, a precursor for coenzyme A, may reflect shifts in oxidative stress responses (Sang *et al.* 2022), and increased tyrosine, a catecholamine precursor, suggests potential changes in neurotransmitter biosynthesis with implications for fetal programming (Garabal *et al.* 1988). Notably, similar metabolic alterations have been reported in placentas from spontaneous preterm birth, where arginine metabolism, creatine metabolism and nucleotide turnover were among the most affected pathways (Cifkova *et al.* 2024). In the fetal brain, inflammation was associated with increased levels of aspartic acid, betaine, carnitine derivatives (Car 2:0 and carnitine), creatine and uracil. Elevated aspartic acid, a key excitatory neurotransmitter and precursor for N-acetylaspartate (NAA), suggests potential disruptions in neuronal signaling (Moffett *et al.* 2007). Higher betaine levels indicate altered methylation processes, which may influence epigenetic programming in the developing brain (Steiger *et al.* 2025). Increased carnitine reflects shifts in energy metabolism (Flanagan *et al.* 2010), while elevated uracil suggests enhanced RNA turnover, possibly as a response to cellular stress (Fan *et al.* 2002). Further studies are needed to evaluate the significance of these additional metabolites as they may offer new insights into the broader impact of intraamniotic inflammation.

The primary strengths of this study include the use of a sophisticated, minimally invasive technique to induce intraamniotic inflammation, which effectively reduces animal stress and adheres to the 3R principles of replacement, reduction and refinement in animal research. Notably, studies in rhesus macaques, sheep and rodents show that intra-amniotic LPS injections primarily affect the fetal environment, causing localized inflammation without significant maternal systemic response or preterm labor (Kallapur *et al.* 2014, Schmidt *et al.* 2016, Stranik *et al.* 2022). In addition, the Wistar rat serves as an ideal model for studying placental function, particularly in the context of TRP metabolism (Badawy 2015). Furthermore, the application of advanced LC/MS techniques combined with unbiased analysis allowed for the identification of a broad range of metabolites, providing a comprehensive understanding of the metabolic alterations induced by intraamniotic inflammation.

A limitation of this study is the 24 h timing of sample collection. Intrauterine inflammation may induce time-dependent metabolic changes, as previous research has shown that the inflammatory response leads to significant but transient alterations in metabolic profiles (Brown *et al.* 2017). Consequently, our 24 h collection point may have missed some transient effects, highlighting the importance of sample timing and the need for time-dependent studies to fully capture these dynamics. In addition, we used whole-tissue homogenates for both the placenta and fetal brain, which precludes the identification of region-specific metabolic changes. This approach, while providing an overview of global alterations, does not allow for the localization of enzymatic or metabolic shifts within distinct placental zones or brain regions. Future studies employing spatially resolved techniques such as tissue sectioning or *in situ* hybridization would help refine these findings. Another limitation is the lack of fetal sex differentiation, as male and female fetuses may respond differently to inflammation. Without sex-specific analysis, potential differences in TRP metabolism and inflammatory responses remain unassessed. Finally, we were unable to directly measure QUIN, a key neurotoxic metabolite in the KYN pathway. Without direct quantification, our conclusions regarding its potential increase rely solely on the gene expression changes of its synthesizing enzymes. Future work incorporating targeted metabolomics would provide a more comprehensive assessment of QUIN dynamics under inflammatory conditions.

**Figure 7:**
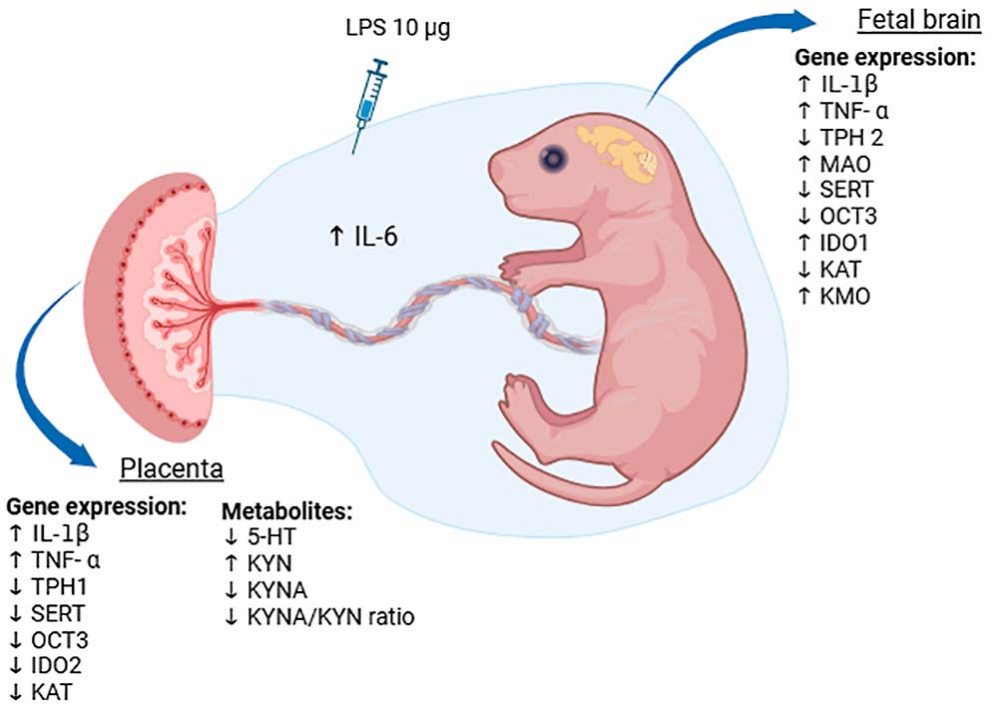
Schematic illustration of the effects of intraamniotic LPS exposure on the TRP metabolic pathways in the placenta and fetal brain. Created in BioRender.com.

In conclusion, this study offers profound insights into the intricate interplay between intraamniotic inflammation, placental function and fetal brain development. Utilizing advanced LC/MS technique and PCR analysis, we delineated significant shifts in gene expression and metabolite levels within the placenta and fetal brain, elucidating the substantial impact of LPS-induced intrauterine inflammation on tryptophan metabolism (Fig. 7). Notably, our findings reveal a potential shift towards neurotoxicity, evidenced by the altered balance of neuroactive metabolites such as KYNA, which is consistent with our previous studies in the human placenta (Abad *et al.* 2024, Cifkova *et al.* 2024).

These findings underscore the critical role of TRP metabolism in mediating the effects of prenatal inflammation on neurodevelopment. However, the exact mechanisms linking these metabolic changes to neurodevelopmental disorders remain to be fully elucidated. Further research is essential to unravel these molecular pathways and understand their long-term impact on fetal brain development. This knowledge could facilitate the way for new therapeutic strategies aimed at mitigating the adverse effects of intrauterine inflammation and safeguarding neurodevelopmental outcomes.

## Supplementary materials



## Declaration of interest

The authors declare that there is no conflict of interest that could be perceived as prejudicing the impartiality of the research reported.

## Funding

Czech Science Foundation (22-08045S) and New Technologies for Translational Research in Pharmaceutical Sciences/NETPHARM, project ID CZ.02.01.01/00/22_008/0004607, co-funded by the European Union. Metabolomic analysis was funded by the Czech Science Foundation (22-13967S).

## Author contribution statement

CA, IM and FS participated in the conception and design of the study. CA, IM, EC, RP, FK, RK, MS, ML, MK, JS, AS and FS participated in the acquisition, analysis, interpretation and critical discussion of the data. CA and FS wrote the article. All the authors contributed to the scientific discussions, revised the article and approved the final draft.
